# Physiotherapy Approach in Transradial Amputation Following the Sequelae of Electric Burn: A Case Report

**DOI:** 10.7759/cureus.51731

**Published:** 2024-01-06

**Authors:** Shraddha S Kochar, Vrushali Athawale, Tejaswini Fating

**Affiliations:** 1 Community Health Physiotherapy, Ravi Nair Physiotherapy College, Datta Meghe Institute of Higher Education and Research, Wardha, IND

**Keywords:** treatment, case report, wound debridement, amputation, burn, electrical shock, physiotherapy

## Abstract

A considerable amount of mortality and morbidity has been attributed to burn injuries. Because of expanding industrial development, greater consumption of electricity, and electric appliances at home, the number of burns caused by electricity is expanding. When it pertains to addressing burn injuries, therapeutic pursuits are necessary. The 18-year-old female patient in this case reported a history of burns due to electrical shock to her left upper and lower limbs along with her right foot. She came to the hospital, and after necessary investigations and examination, she was suggested for below elbow amputation or transradial amputation with debridement of the wound over the left axilla, foot, and right foot. Following the surgical procedure, the patient had pain, reduced muscle strength, limited joint mobility, and edema. For further rehabilitation, community health physiotherapy was advised. Routine physiotherapy treatment was provided to the patient for fifteen days. We report that after surgery, the effects of physical therapy showed decreased pain intensity, enhanced range of motion (ROM) of the affected and peripheral joints, and improved strength in adjacent muscles.

## Introduction

Heat, gas steam, chemicals, electricity, radiation, and friction are some of the various kinds of injuries to the skin that may end up in burns. These injuries may occur at a variety of temperatures, from the lowest frostbite-related injuries to the highest electrical-related injuries, and as a consequence, the burns develop a coagulative altering of proteins [1]. The growth in industrial development, consumption of electricity, and usage of electrical appliances in households are contributing variables to an increase in the number of burn cases due to electricity [2]. Skin cell proteins denature and coagulate in response to burns, prompting thrombosis to develop in the blood vessels [3]. In addition to the potentially difficult-to-manage effects of multiple organ failures, metabolic changes, an inflammatory and immunological response, and distributive shock, injuries caused by burns, especially severe ones, also have an immediate effect on the person's health as a whole and physical as well as mental health [4]. Electrical burns are a frequent risk at home and work [5]. Electrical injuries are different because they might result in significant damage to the tissue under the skin's surface in addition to the area of the wound itself [6]. Depending on the thickness of the damaged skin, there are variations in burn injury severity. Mild or minor, moderate, and significant or severe are the three main classifications determined by the burn percentage [7]. Variations in voltage, current strength, resistance, current path, duration of contact, and the overall state of the human body all impact how electricity damages the body [8]. Electric burns may happen due to unintentional contact with low-voltage current (less than 1000 volts), high-voltage current (greater than 1000 volts), and sometimes through voltaic arcs and lightning [9]. High-voltage current damages tissues that transmit electrical energy throughout the body, additionally causing injury directly at the point of contact [10]. The bone is the structure that accumulates the most tremendous amount of heat when a high-voltage current is passed through it because it possesses the highest electrical resistance [11].

Amputation is defined as the surgical removal of the entire or a portion of a digit or extremity through a joint or bone [12]. Despite the best care, full-thickness circumferential burns and electrical burns could ultimately necessitate amputation [13]. The most devastating consequence of burn injuries is the loss of a limb; therefore, opting to amputate a severely burned limb is essential for increasing the patient's chances of survival and minimizing morbidity [14]. With an incidence of as much as 68 percent, burns caused by electricity have a direct connection to an increased probability of amputation [15]. Problems regarding burn intensity, rate of infection, and mechanism of injury have all been associated with delayed amputations. Amputations additionally cause long-term socioeconomic consequences for patients and their families, plus increasing rehabilitation procedures [16]. Maintaining range of motion (ROM), decreasing contracture formation, enhancing function, and fostering psychological well-being and social integration are all made achievable by physiotherapy rehabilitation, which has become a vital component of burn treatment [17]. Individuals with burns are more susceptible to becoming bedridden from ongoing pain medication, drowsiness, bandaging, and dressing of wounds; hence, it may be advisable for physicians to recommend physiotherapy [18].

## Case presentation

Patient information

An 18-year-old female arrived at the casualty with a history of burns by high-voltage electric wirings. She was alright ten days back when, while coming downstairs from the terrace, she accidentally touched a high-voltage current line with her bare hand and fell prone. Immediately, she was taken to the government hospital for primary treatment through ambulance, where a blood transfusion of two units was given for two days. Due to insufficient treatment facilities at the government hospital, she was referred and taken to a tertiary care hospital. The patient had sustained electrical burn injuries on both the left upper extremity and lower extremity, along with the right foot. After examinations, which showed maggots and dead necrotic tissues over the left hand up to mid-forearm, the patient was suggested for amputation of the left upper limb (below the elbow/transradial amputation) and skin grafting as time proceeds along with wound management. She did not have any history of head injury, loss of consciousness, chest trauma, or convulsion. The patient was observed post-operatively for two days in the intensive care unit (ICU). The patient received a referral to community health physiotherapy for further treatment following the surgery since her primary symptoms were pain, reduced joint movement, diminished muscular strength, and edema.

Clinical findings

Before performing the examination, the individual's both written and verbal consent was obtained. Before the surgical procedure, a local examination was done, which showed subcutaneous tissue exposed in the left axillary region along with blackening of the skin of the left hand and axilla. Tendons and bone were exposed in the left forearm, with edema in the left arm and black eschar in the left forearm. On examination of the left leg, there was a superficial burn in the calf region with second-degree burns in the anterior aspect of the leg with the absence of pulsation. The sensory and motor examinations revealed no impairment. Maggots and dead necrotic tissues over the left hand up to mid-forearm were seen on observation. Therefore, the patient was advised for transradial amputation of the left side. There was a fourth-degree burn of the left hand up to the mid-forearm and a second-degree burn above that. In order to calculate the total body surface area involved in burned individuals, trauma and emergency medicine experts utilize Wallace's rule of nine. Wallace's rule of nine was used to compute the percentage of the whole surface of the burned body area, which in this case was 18%. After the operation, an examination showed the upper and lower joints had reduced ROM. The patient reported a 7.9/10 rating for pain severity on the Visual Analogue Scale (VAS). It also showed a notable reduction in muscle strength.

Investigatory findings

**Figure 1:**
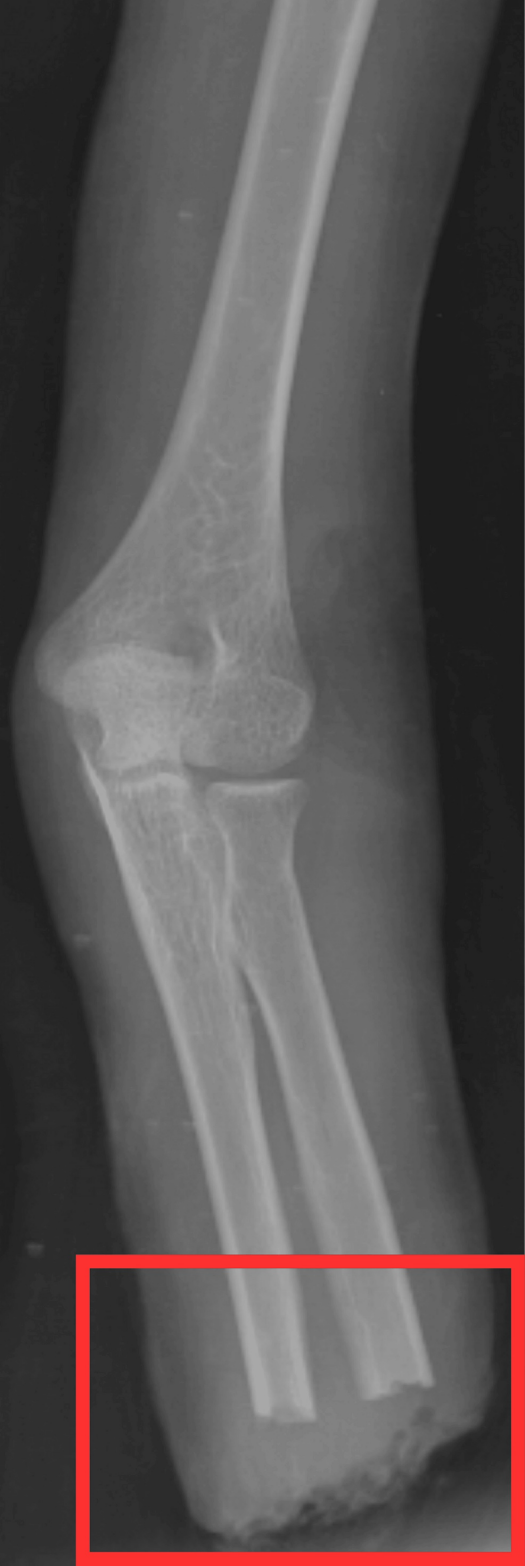
Post-operative X-ray of left amputated upper limb The red rectangle shows the transradial amputation of the right upper limb.

Investigations were performed, which included a complete blood count (CBC) that revealed reduced hemoglobin (Hb), sodium, and potassium levels, which is shown in Table 1; on microbiological studies, the blood culture specimen showed growth of pseudomonas species. After the amputation was done, an X-ray was taken, which is shown in Figure 1.

**Table 1:** Complete blood count Hb - hemoglobin; g/dL - grams per deciliter; mmol/L - millimoles per liter

Blood investigation	Value	Normal values
Hb	6.8 g/dL	For males: 14-18 g/dL; for females: 12-16 g/dL
Sodium ion	126 mmol/L	136-145 mmol/L
Potassium ion	3.1 mmol/L	3.6-5.2 mmol/L
Magnesium ion	1.2 mmol/L	0.4-0.6 mmol/L
Phosphorous ion	1.52 mmol/L	0.097-1.45 mmol/L

Surgical procedure

**Figure 2:**
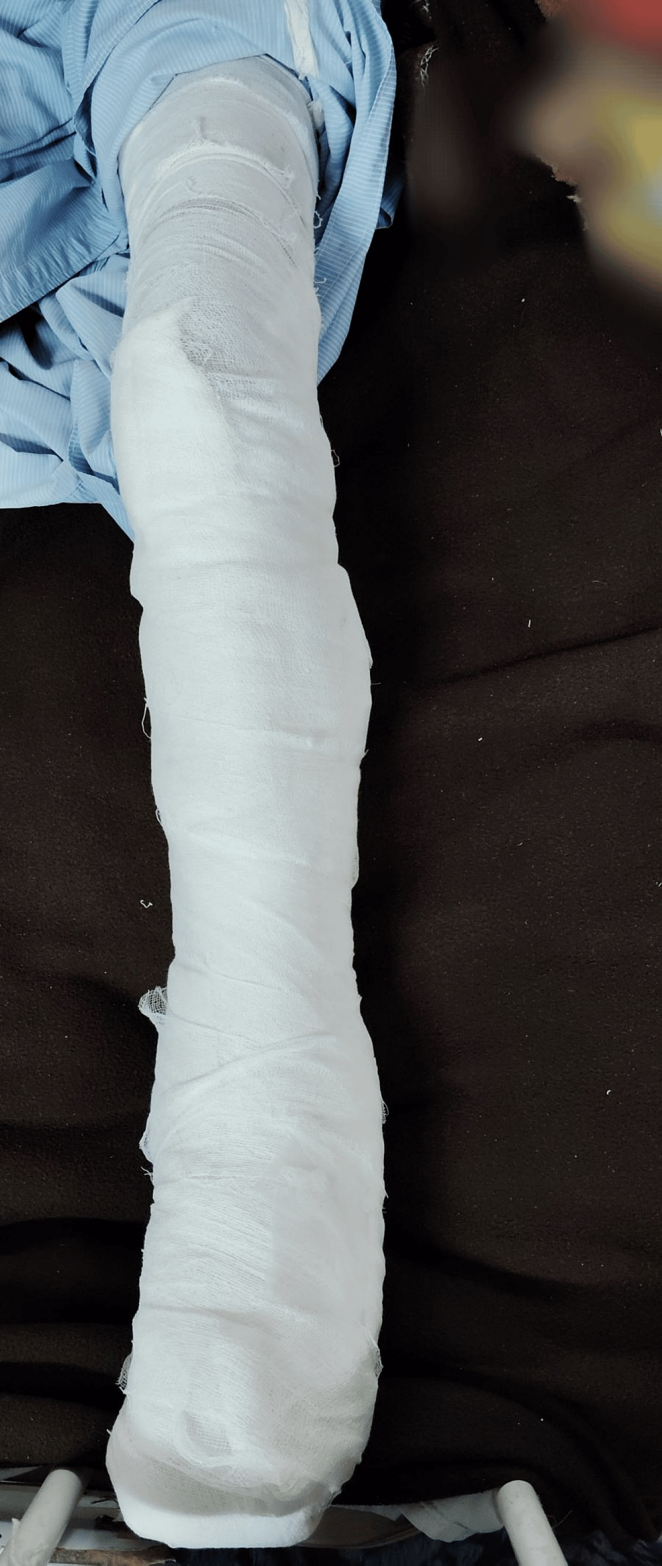
Left lower limb post-operatively

**Figure 3:**
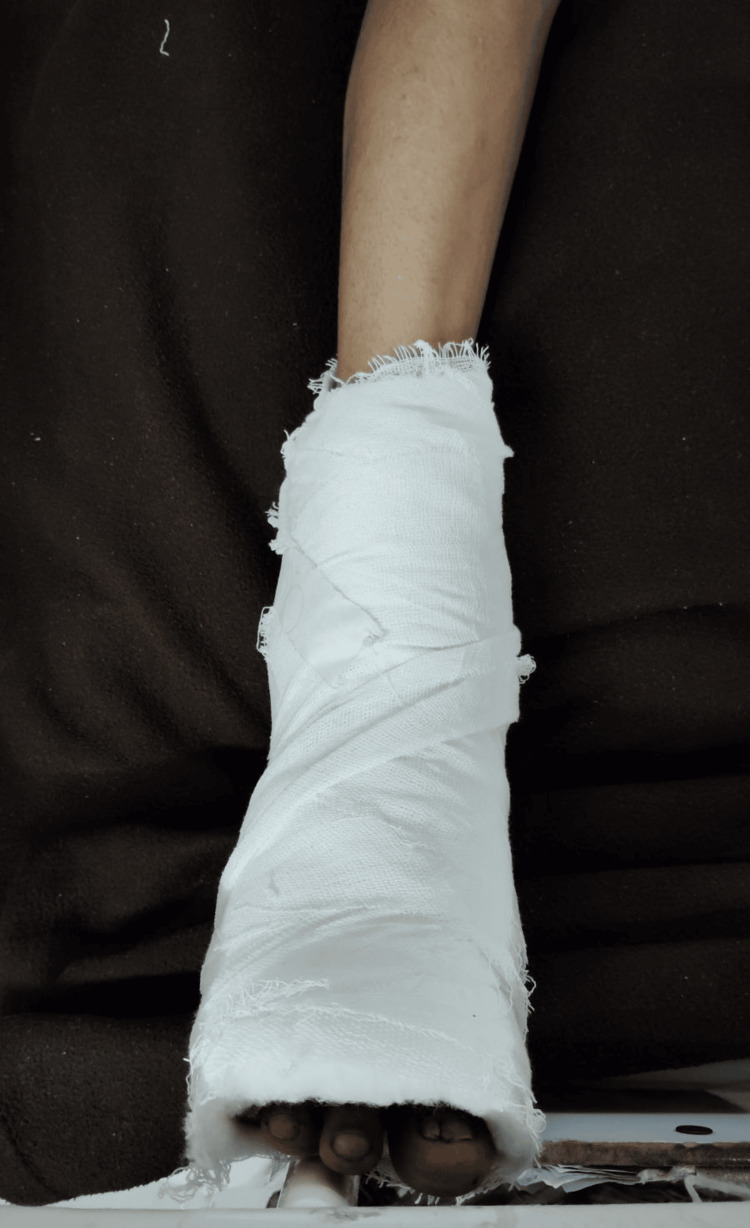
The right lower foot post-operatively

Transradial amputation with debridement of the wound over the left axilla, foot, and right foot was done. The incision was taken at the distal forearm over the gangrenous part of the left forearm. Muscles were clamped, cut, and ligated. Then, the periosteum of the ulna and radius was elevated, and bone was cut with the help of a jiggly saw. Further necrotic muscle and soft tissue were removed. Debridement of the wound over the left foot was done, the slough was removed, and healthy bleeding tissue was observed. This similar procedure was repeated over the left cubital fossa and the left axilla. Figures 2-3 show the patient's limbs after the surgical procedure.

Physiotherapeutic intervention

**Figure 4:**
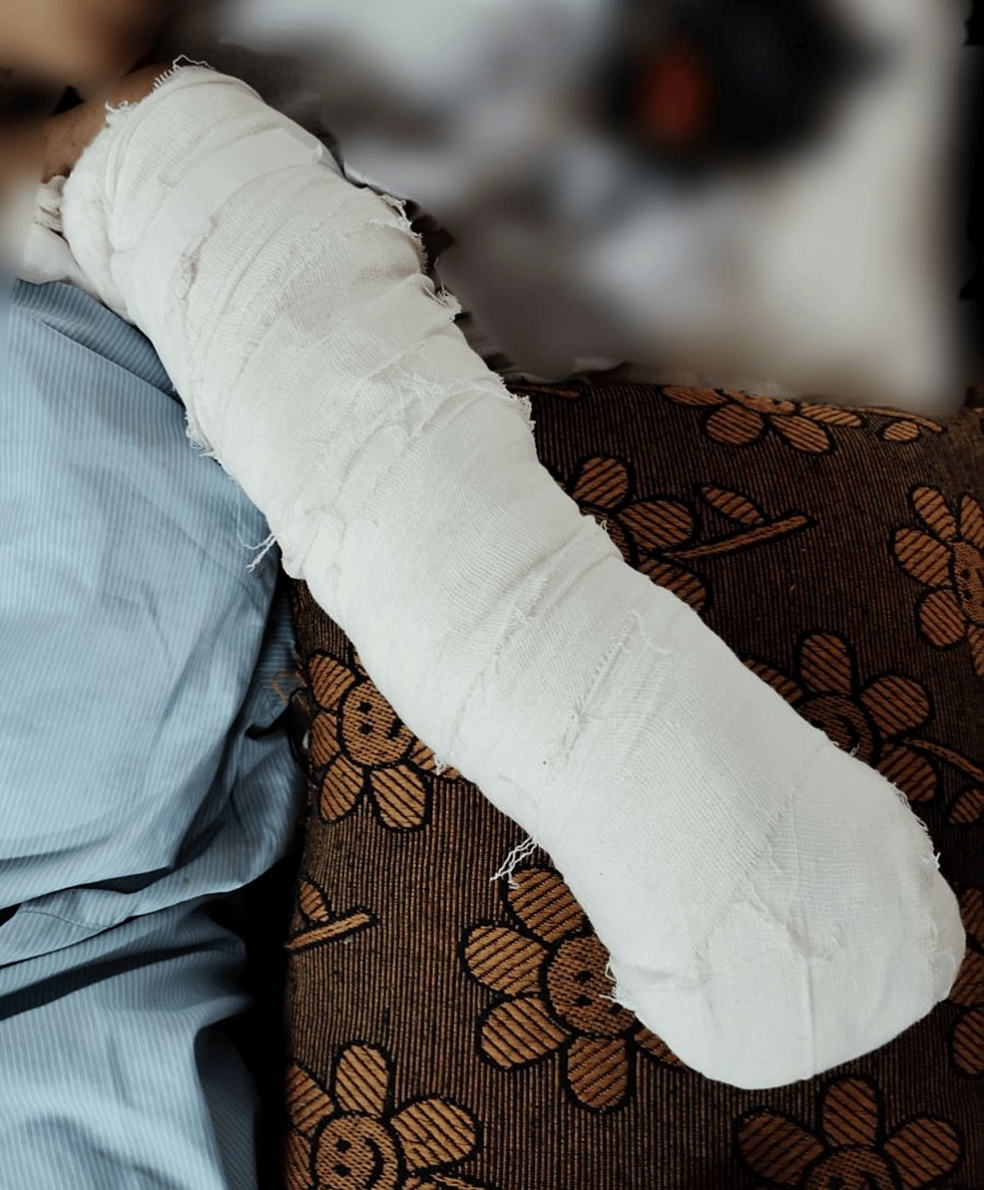
Limb kept in elevation

Table 2 shows the physiotherapy rehabilitation protocol which was given to the patient. Figure 4 shows the patient's amputated limb kept in elevation to reduce the edema with the pillow underneath.

**Table 2:** Physiotherapy rehabilitation ROM - range of motion; AAROM - active assisted range of motion; AROM - active range of motion; reps - repetition; SLR - straight leg raise

Goals	Intervention	Rehabilitation program
To educate the patient and her family	-	The patient obtained a detailed description of the issue she was experiencing, in addition to the importance and benefits of physical therapy treatment. It was additionally made clear to her and her family how the therapy would prevent consequences while also improving her quality of life (QoL) by enabling her to walk, climb stairs, and perform other daily tasks.
To reduce pain and stimulate the involvement of the patient	Music therapy	Distracting the patient from their perception of pain via music therapy during a physical therapy session enhanced their cooperation and participation.
To improve the ROM of the left shoulder, hip, knee, and ankle joints and the right ankle joint	AAROM and AROM exercises	AAROM exercise was performed for all the mentioned joints (10 reps x 1 set) twice a day for nine days, which was followed by AROM exercise (10 reps x 2 sets) thrice a day for six days till the pain-free range.
To improve the strength of muscles	Isometric exercises	Isometric exercises were given to both the lower limbs quadriceps, hamstring, and gluteal muscles. An assistive SLR was also initiated.
To control edema	Limb elevation	For the acute phase of the burn wound, limb elevation was given to the patient by keeping her limbs in an elevated position with the support of a pillow underneath.
To enhance the upper limb mobility	Upper limb mobility exercise	A thoracic expansion exercise was given to the patient in which the patient was told to inhale through her nose while taking both her upper limbs up (that is full shoulder flexion), hold the breath for about two seconds, and then exhale the breath out through the mouth by taking the limbs to their original position. This exercise improved her upper limb mobility as well as chest expansion.
To increase functional independence	Early mobilization	Ambulation was started on the third day post-operatively. Initially, the patient was ambulated around the bed only (with assistance); after one week of surgery, hall ambulation was initiated. Early mobilization was done to avoid the occurrence of any secondary complications.

Follow-up and outcome measures

After 15 days of therapy, a follow-up was conducted. Table 3 shows the pre- and post-rehabilitation ROM findings. Outcome measures include pain grading through the VAS, and to analyze the ability of an individual to carry out activities of daily living (ADLs), the Barthel index was taken, which is depicted in Table 4.

**Table 3:** ROM of the left upper extremity, the left and right lower extremity AROM - active range of motion; ROM - range of motion

Joint movement (AROM)	Left upper extremity: day 1	Left upper extremity: day 15
Shoulder flexion	0^o^-100^o^	0^o^-170^o^
Extension	0^o^-15^o^	0^o^-25^o^
Abduction	0^o^-90^o^	0^o^-130^o^
	Left lower extremity: day 1	Left lower extremity: day 15
Hip flexion	0^o^-50^o^	0^o^-75^o^
Abduction	0^o^-20^o^	0^o^-35^o^
Adduction	0^o^-10^o^	0^o^-22^o^
Knee flexion	0^o^-45^o^	0^o^-72^o^
Ankle dorsiflexion	0^o^-5^o^	0^o^-15^o^
Plantarflexion	0^o^-10^o^	0^o^-25^o^
	Right lower extremity: day 1	Right lower extremity: day 15
Ankle dorsiflexion	0^o^-8^o^	0^o^-18^o^
Plantarflexion	0^o^-14^o^	0^o^-29^o^

**Table 4:** Outcome measure VAS - Visual Analogue Scale Barthel index interpretation: 80-100 - the patient is able to live independently, 60-79 - minimally dependent, 40-49 - partially dependent, 20-39 - very dependent, less than 20 - totally dependent

Outcome measures	Pre-rehabilitation	Post-rehabilitation
VAS	7.9/10	3.2/10
Barthel index score	25/100 (very dependent)	78/100 (minimally dependent)

## Discussion

Burns are referred to as wounds that cause coagulation-induced tissue necrosis. The increasing number of cases is the consequence of industrialization and the complexities of contemporary life [19]. High-voltage current damages tissues that conduct the electricity around the body in addition to causing direct injury at its point of contact. The total amount of electric current moving through is directly proportional to the extent of damage to tissue developing between the electric current's location of entry and its point of exit. Further, the tissues' resistance determines the extent to which damage is caused. In managing burns due to electrical shock, early physiotherapy plays a vital role in reducing secondary complications, improving the patient's quality of life (QoL), and enhancing the patient's functional independence [9].

According to the Çınar et al., individuals who have suffered significant injuries from burns are encouraged to start their physical therapy regimen as quickly as possible. In conjunction with standard medical treatments, a physical therapy program must be undertaken to minimize pain, boost joint ROM, rebuild the strength of the muscles surrounding the involved joints, and ultimately enhance the individual's overall QoL [19]. Physiotherapists who manage those suffering from injuries caused by burns may encounter specific problems and difficulties, as stated by Dunpath et al., since they have to reduce the cause of pain to a person who is already traumatized, nervous, and afraid for the individual to recover from their injuries. Since burn injuries often result in psychological reactions requiring support or psychological therapy, the acute stage of therapy is just as significant for the individual receiving treatment along with the rehabilitation expert [17]. Pain treatment in patients with burns is a continually changing challenge for the burn care team, as stated by Rohilla et al. Pain management in burn patients is associated with physiological and psychological implications. Music therapy can help reduce the level of pain and anxiety and encourage the patient to perform the exercise regime [20]. In this case, we observed the application of various treatment approaches, including music therapy, isometric exercises, active assistive and active movements, limb elevation, thoracic expansion exercise, and early ambulation. These strategies resulted in the accomplishment of a functional ROM and an improvement in muscle strength within 15 days of the physical therapy rehabilitation.

## Conclusions

Early rehabilitation with physical therapy is beneficial and has considerably enhanced the ability of the individual to participate in routine tasks following an electric burn injury. The main objective of the physiotherapy approach is to prevent burning injuries, which lead to subsequent complications that conflict with the individual's daily activities. In the patient's finest interests, this case report illustrates a carefully thought-out, encompassing strategy for treatment.
